# Deconvoluting Wavelengths Leading to Fluorescent Light Induced Inflammation and Cellular Stress in Zebrafish (*Danio rerio*)

**DOI:** 10.1038/s41598-020-59502-5

**Published:** 2020-02-24

**Authors:** Mikki Boswell, William Boswell, Yuan Lu, Markita Savage, Ronald B. Walter

**Affiliations:** The Xiphophorus Genetic Stock Center, Department of Chemistry and Biochemistry, Texas State University, San Marcos, TX 78666 USA

**Keywords:** Computational biology and bioinformatics, Gene regulatory networks

## Abstract

Fluorescent light (FL) has been shown to induce a cellular immune and inflammatory response that is conserved over 450 MY of evolutionary divergence and among vertebrates having drastically different lifestyles such as *Mus musculus*, *Danio rerio*, *Oryzias latipes* and *Xiphophorus maculatus*. This surprising finding of an inflammation and immune response to FL not only holds for direct light receiving organs (skin) but is also observed within internal organs (brain and liver). Light responsive genetic circuitry initiated by the *IL1B* regulator induces a highly conserved acute phase response in each organ assessed for all of biological models surveyed to date; however, the specific light wavelengths triggering this response have yet to be determined so investigation of mechanisms and/or light specific molecule(s) leading to this response are difficult to assess. To understand how specific light wavelengths are received in both external and internal organs, zebrafish were exposed to specific 50 nm light wavebands spanning the visible spectrum from 300–600 nm and the genetic responses to each waveband exposure were assessed. Surprisingly, the induced cellular stress response previously observed following FL exposure is not triggered by the lower “damaging” wavelengths of light (UVB and UVA from 300–400 nm) but instead is maximally induced by higher wavelengths ranging from 450–500 nm in skin to 500–600 nm in both brain and liver).

## Introduction

Although both research animals and humans are spending increasing amounts of time indoors and under commonly used fluorescent light bulbs, little is known about potential health and genetic effects due to use of this type of artificial light. Recently, the genetic effects of fluorescent light (FL, “cool” white, 4100 K) exposure were investigated and surprisingly this light source was found to induce a highly conserved inflammatory and cellular immune genetic response in three biomedical fish models and the hairless mouse^1,2^. Thus, the conserved FL inflammatory/immune response spans ~450 MY of evolution, is observed in both a direct light receiving organ, skin, as well as in brain and liver. The skin and the brain showed up-regulation of the inflammation/immune response in all animals tested. However, in the liver, even though the same genetic pathways and upstream regulators were modulated in both aquatic and terrestrial vertebrates, the mouse liver exhibited a suppressed response rather than up-regulation as in the fishes. This may be due to the nocturnal lifestyle of the mouse and the liver’s ability to adjust its gene expression patterns to metabolic cycles, rather than to light regulation. Regardless, the interesting genetic conservation of response to FL is reported in vertebrates spanning different environmental niches (new world tropical versus old world marsh lands), reproductive mechanism (oviparous versus viviparous), and lifestyle (diurnal versus nocturnal). Thus, we speculate this non-circadian based light response is deeply imbedded within the vertebrate genome.

**Figure 1:**
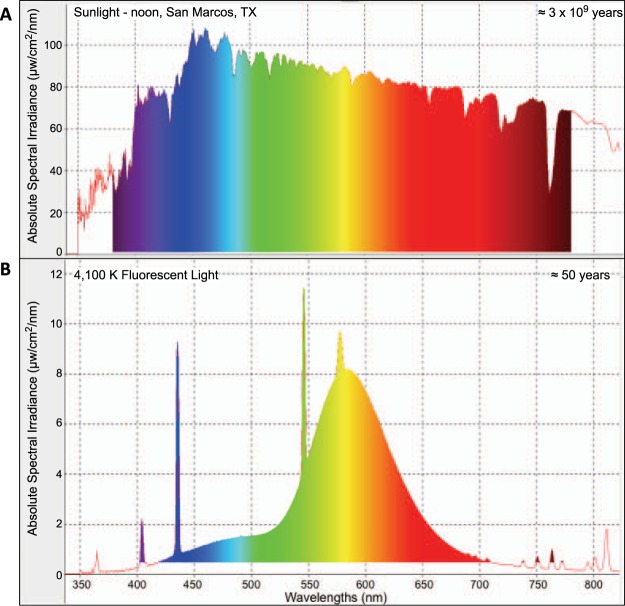
Spectral distribution of natural sunlight in San Marcos, TX at noon (top, **A**) and Phillips 4100 K cool white bulbs used for the FL exposures (bottom, **B**).

Fluorescent light (FL) is comprised of wavelengths spanning from ~350–800 nm and overall shows many peaks and valleys of specific wavelength intensities (Fig. 1b). In contrast, the solar spectrum over these wavelength regions is both intense and with all wavelengths exhibiting fairy consistent intensities. The uniform spectral distribution of sunlight may suggest that light responsive genetic circuitry has evolved whereby receipt of each wavelength by the organism triggers different biological cues to produce proper genetic modulation leading to proper adaptive behavior as optimized for the specific environmental niche of each organism. Therefore, one might expect the vastly narrowed FL spectrum, compared to sunlight (Fig. 1a), may have the potential to trigger different and abnormal genetic responses that could lead to maladaptive biological behaviors at the molecular, and perhaps organismal levels. Since light responsive genetic circuitry is well conserved, it is important to determine which wavelengths throughout the FL spectral distribution may cause the observed genetic responses in inflammatory and immune pathways.

In addition to FL, we have reported genetic profiling results of *Xiphophorus maculatus* (platyfish) exposed to each 50 nm wavelength region between 350–600 nm (i.e., 350–400, 400–450, … etc. to 600 nm^3,4^). Interestingly we observed the two most genetically active regions spanned 350–400 nm (i.e., UVA region) and 500–550 nm (i.e., green). These two optically active regions had very different genetic signatures that were characterized by the suppression of cell cycle progression and induction of cellular DNA damage repair. It was also reported that two known DNA repair regulators could be induced by independent wavelength regions (*ATM* following 350–400 nm and *ATR* following 500–550 nm); this finding suggested one might use discrete waveband exposure as a tool to regulate and control key genetic pathways^4^. Detailed analyses of RNAseq data from the 50 nm waveband exposures showed that each 50 nm waveband induced and/or suppressed specific genetic pathways. It was also shown that different waveband exposures could up- or down-regulate the same genetic pathways, a phenomenon we termed *waveband specific gene regulation*. For example, the epidermal growth factor receptor (EGFR) pathway can be up regulated by exposure to 450–500 nm light, but transcription of genes in this pathway is substantially down-regulated after exposure to 520–530 nm light^3^. Further, it was observed that, in terms of genetic response, some wavelengths were dominant to others. Platyfish exposed to 510–520 nm light, then immediately followed by exposure to 350–360 nm light, displayed the genetic response that mimicked only the 510–520 nm exposure; however, when the exposure sequence was reversed (i.e., exposure to 350–360 nm light followed by 510–520 nm light) the genetic effect was muted, yet more closely reflected the 510–520 nm exposure results. This indicated for the first time that higher, more red shifted wavelengths of light were potentially dominant in their effect on genetic modulation than are lower wavelengths of light.

These experiments^1–4^ and others^5–7^ were the first to characterize the genetic response of an intact animal to various light sources and/or specific wavelengths. However, they do not directly address the question of how FL induces highly conserved inflammatory and cellular immune responses initiated by the *IL1B* regulator, in both a direct light receiving organ and internal organs. Herein, we detail results from experiments using adult male zebrafish exposed to either FL or discrete 50 nm wavebands across the FL spectrum (300–600 nm) that have allowed us to carefully trace the genetic regulators and pathways of the inflammation and immune responses to specific wavebands of light in each organ (skin, brain and liver). We present results showing skin induces a cellular immune and inflammatory response starting at 400 nm and peaking after 450–500 nm light exposure while brain and liver are red shifted showing this genetic response at 500–550 nm and 550–600 nm, respectively. The highly conserved response observed in three organs suggests there is direct reception within internal organs of specific light wavelengths and supports previous findings that higher red-shifted wavelengths are genetically dominant to potentially harmful UVA/UVB wavelengths of light.

## Results

### Sequencing profiles

Following FL exposure, zebrafish skin modulated significantly more genes than after exposure of any of the six 50 nm wavebands (Table 1). However, both brain and liver modulated more genes following exposure to each of the 50 nm wavebands than following FL exposure. On average, 50% of the DEGs determined as significantly differentially expressed were successfully mapped and analyzed by IPA.

**Table 1:** The number of differentially regulated genes was determined following EdgeR (column 3), after sham removal (column 4), after assignment of human homolog genes (column 5) and after IPA analysis (column 6). Differentially expressed genes for FL exposed samples. Total modulated genes are the output file from EdgeR (column 2) that had a log_2_(fold change) ≥ |2.0| and a (p-adj < 0.05). All fish Ensembl IDs were converted to HUGO IDs (column 5) for IPA analysis. HUGO IDs were then imported and mapped by Qiagen’s IPA software for functional and pathway analysis (column 6).

Organ	Waveband	DEG	Sham Removed	HUGO ID	IPA Mapped
Skin	FL	801	759	671	615
	300–350	363	312	182	158
	350–400	352	214	177	162
	400–450	472	433	223	203
	450–500	301	280	149	137
	500–550	172	150	81	79
	550–600	285	249	152	140
Brain	FL	71	66	55	48
	300–350	237	156	91	89
	350–400	373	258	162	150
	400–450	503	419	223	205
	450–500	337	253	146	137
	500–550	345	259	174	156
	550–600	406	315	200	178
Liver	FL	83	60	52	47
	300–350	278	241	167	156
	350–400	253	218	166	156
	400–450	177	159	110	105
	450–500	111	92	58	56
	500–550	253	236	166	161
	550–600	266	227	158	145

**Figure 2:**
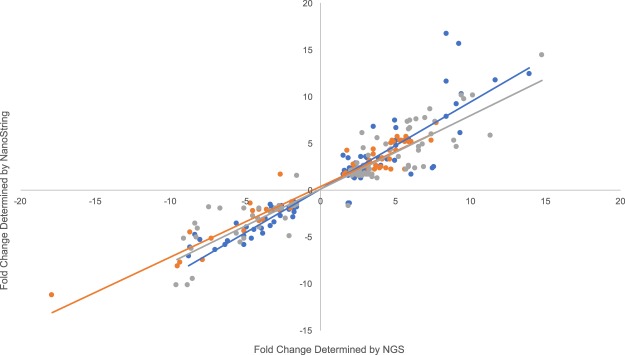
NanoString nCounter technology was used to confirm the fold changes determined using EdgeR in skin (blue), brain (orange) and liver (gray).

Differentially expressed genes were confirmed using NanoString’s nCounter assay with a custom oligo panel (Table S1). This independent technology tested 84, 68, and 86 targets in skin, brain and liver with R^2^ values equal to 0.85, 0.93 and 0.85 respectively (Fig. 2). Out of the genes tested, only five targets did not confirm in direction (two in skin, one in brain and two in liver).

### Waveband responses in skin

**Figure 3:**
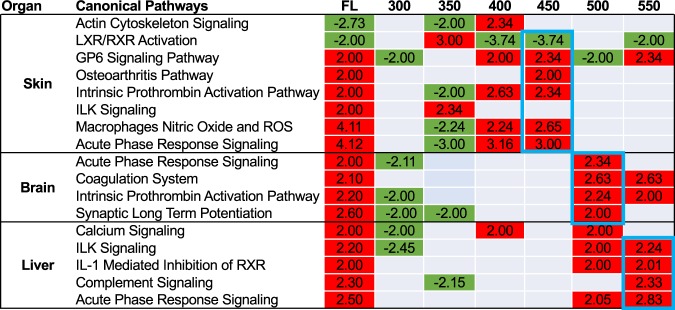
FL modulated pathways in zebrafish skin, brain and liver consistent with an up-regulation of the immune and inflammatory response. 50 nm regions were surveyed from 300–600 nm to determine which specific wavelengths were responsible for this response. Red indicates specific canonical pathways as determined by IPA that had a z-score of > 2 and green represent pathways with a z-score < 2. The numbers inside of each box represent the IPA determined z-score of modulation. Both 400–450 and 450–500 nm reflected the FL response in skin; 500–550 nm in brain and 550–600 nm in liver. The primary waveband mimicking the FL response is highlighted in blue for each organ. To see the number of genes represented in each pathway see Fig. S1.

As previously reported, the FL response in zebrafish skin, brain and liver is controlled through the *IL1B* regulator and conserved up-modulation of the Acute Phase Response signaling pathway (APR^1,2^). This pathway and others promote a conserved inflammatory and immune response throughout the animal. Following exposure to 50 nm wavebands, the most direct light receiving organ, skin, up modulated the APR pathway after 400–450 and 450–500 nm exposures (3.16 and 3.00 fold, respectively). In addition, the skin response in these two wavebands up modulated GP6 signaling (2.00 and 2.34 fold, respectively), Prothrombin activation (2.63 and 2.64 fold), production of Nitric Oxide and ROS in macrophages (2.24 and 2.65 fold) and exhibited suppression of LXR/RXR activation (−3.74 fold in both wavebands). In addition, the 450–500 nm waveband activated the Osteoarthritis pathway (2.00 fold, Fig. 3). The lower wavebands suppressed many of the immune and inflammatory pathways that were activated in the 400–500 nm regions (i.e, mentioned above, and Fig. 3, green) while also up-modulating the LXR/RXR activation pathway.

**Figure 4:**
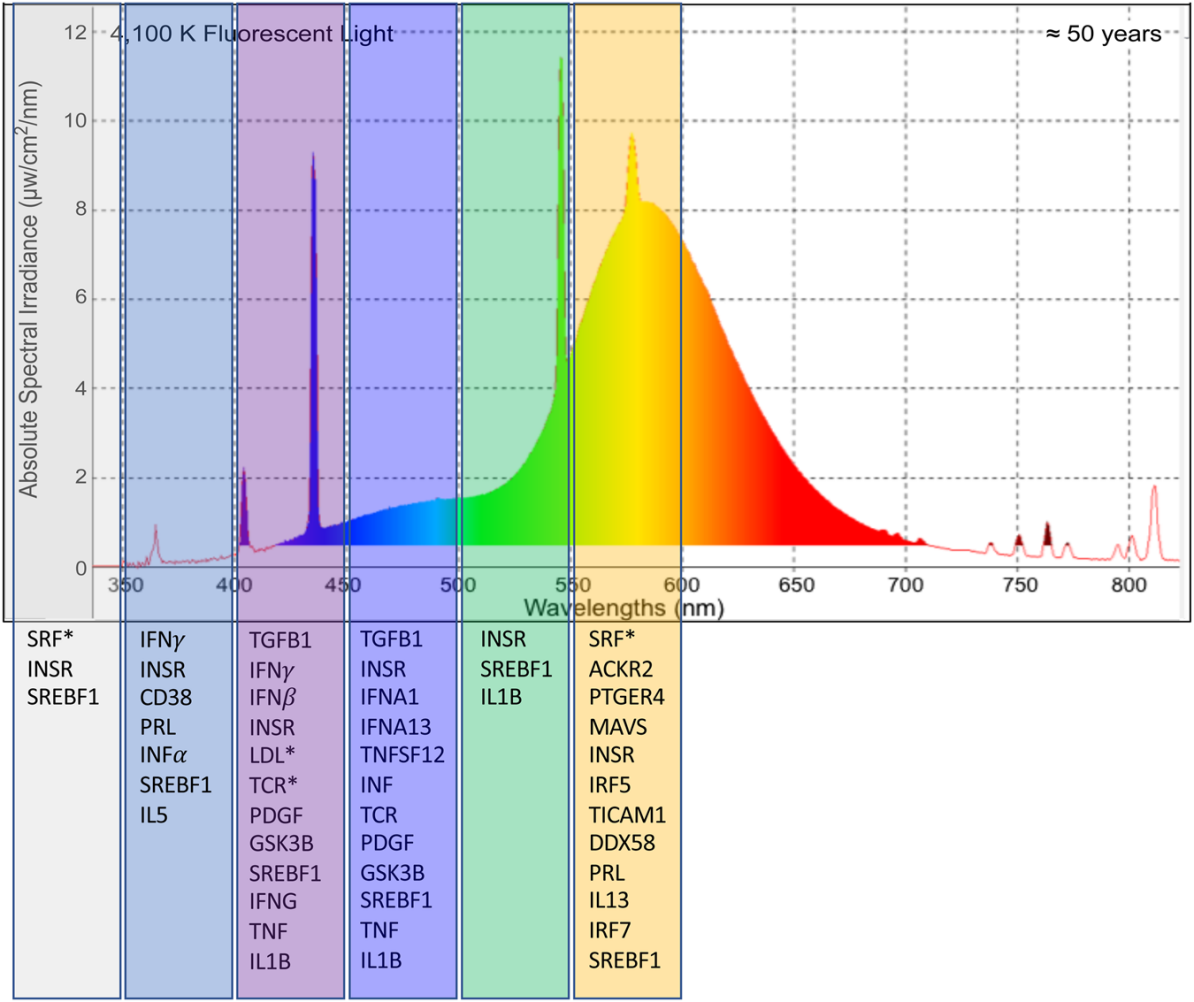
Upstream regulators modulated by discrete 50 nm wavebands that are also modulated by FL. Genes with an * are oppositely modulated following 50 nm exposure compared to the complex FL exposure. We observed both light responsive gene expression (ie. genes modulated in response to all light stimuli; *INSR* and *SREBF1*) and waveband specific gene expression (ie. genes modulated in response to only discrete wavelengths).

FL stimulates 104 upstream regulators that can be categorized into (a) immune and inflammation, (b) cellular proliferation and cellular signaling and (c) motility responses. Individual 50 nm wavebands modulated 28 regulators which were segregated by waveband specific modulation (*IL1B, TNF, CD38*, etc.) or light responsive modulation (*INSR* and *SREBF1*, Fig. 4). Herein, waveband specific modulation refers to specific genes or pathways that are only modulated by specific wavebands of light (or directionally controlled by specific wavebands of light, ie., up-modulated by one waveband and down modulated by another) whereas light responsive modulation refers to genes or pathways that are modulated in response to multiple wavebands or complex light sources. Each waveband also modulated regulators that were not shared with the FL response (Table S2). The single wavelength regulators induced by 400–450 nm light include *IL6, TGFb, STAT3, IFNA2, STAT5B, NOS2, STAT1* and *JAK1/2*. This along with the specific pathways modulated by this waveband indicate that 400–450 nm light is likely responsible for the conserved inflammatory and immune response observed in the skin of zebrafish. In addition, a continuation of this response into the 450–500 nm region likely contributes to responses including oxidative cellular stress and the inflammatory response.

### Waveband responses in brain

In zebrafish brain, FL modulated four pathways including the APR. Following 50 nm waveband exposures, the APR pathway was up modulated following 500–550 nm light (2.34 fold). This pathway along with the Intrinsic prothrombin activation pathway, synaptic long-term potentiation pathway and the coagulation system indicate the conserved immune and inflammatory responses, which is activated by 400–500 nm light in skin, is red shifted about 50 nm in brain and up-modulated by 500–550 nm light (Fig. 3).

**Figure 5:**
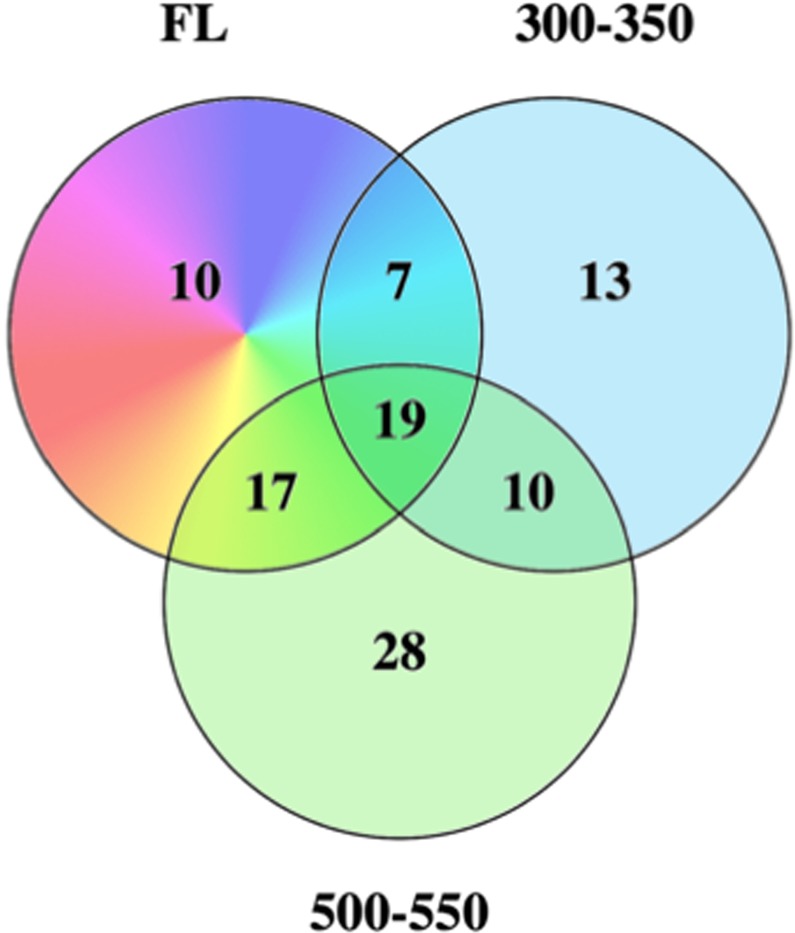
Upstream regulators modulated in FL and the two primary shared wavebands within the FL spectrum. While both regions have significant gene identity sharing with the FL exposed brain samples, the 300–350 nm region oppositely modulated genes compared to FL and the 500–550 nm region modulated genes in the same direction as FL (Table 2).

Analyses of upstream regulators indicates that while cell cycle progression is up-modulated following 450–500 and 550–600 nm exposure, only the 500–550 nm waveband served to up-modulate regulators consistent with the FL induced inflammatory and immune responses (Table S3); including *IL1B* and *TNF*. In total, 26 regulators were modulated in both FL and 300–350 nm light, while 36 were modulated in both FL and 500–550 nm light exposures (Fig. 5). While 300–350 nm shared the second highest percentage of regulators modulated with FL, 69% (18 of the 26 regulators) were modulated in the opposite direction (Table 2). However, following 500–550 nm exposure, all but one regulator, *PPARA*, exhibited modulation in the same direction. Like in skin, both waveband specific and light responsive regulators were identified. Interestingly, the primary regulators responsible for the brain inflammatory response were waveband specific and include; *IL6, IL1B, TNF* and *INFG*. *PPARG* was the only light responsive regulator that was significantly modulated in every light exposure.

**Table 2:** The FL induced regulators were primarily observed following two discrete wavebands of light (300–350 and 500–550 nm).

Upstream Regulators	FL	300–350	500–550
Tgf beta	2.62	−2.86	2.00
TGFB1	2.65	−2.23	2.33
TNF	3.27	−2.02	2.42
MYC	2.00	2.59	2.43
Insulin	2.03	−2.00	2.49
IFNG	2.14	−2.58	2.52
NFE2L2	2.08		2.75
IL10	2.61	−3.03	2.97
Hdac	2.00		2.97
IL4	2.04	−2.88	2.98
STAT1	3.11	−2.91	2.99
PPARA	−2.31	−2.93	3.04
RXRA	2.15		3.06
IL1	3.11	−2.99	3.08
TGFBR2	2.07		3.12
PPARGC1A	2.11	4.15	3.22
ERBB2	2.62		3.31
IL6	3.16		3.34
OSM	2.41	−3.25	3.35
PPARGC1B	2.00		3.41
PPARD	2.10	2.41	3.64
P38 MAPK	2.01		3.64
MEF2C	2.11		3.67
AKT1	2.89		3.67
CREB1	2.41	2.94	3.67
STAT3	3.02		3.71
IL1B	4.12	−2.00	3.85
PPARG	2.00	2.68	3.90
ERK1/2	2.65	−3.71	3.91
MTOR	2.86		3.92
SMAD4	2.59		3.93
PRDM1	2.55		3.97
F2	2.91	−3.07	4.13
CD38	2.10		4.18
FOXA2	2.32		4.19
IL5	2.45		4.60
EGFR	2.61	−3.71	
Pkc(s)	2.05	−3.04	
PRL	2.00	−3.03	
TP53	2.00	−2.18	
SPP1	2.11	2.10	
GATA4	2.61	3.03	
AR	−2.52	3.18	

While both regions have significant gene identity sharing with the FL exposed brain samples (Fig. 5), the 300–350 nm region oppositely modulated genes compared to FL and the 500–550 nm region modulated genes in the same direction as FL. The full list of modulated upstream regulators is presented in Table S3.

### Waveband responses in liver

**Figure 6:**
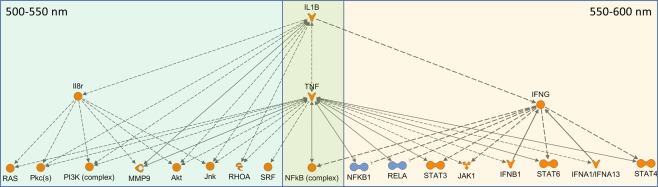
Upstream regulators modulated in liver by both FL and 500–550 nm (left) or 550–600 nm (right). *IL1B, TNF and NFkB* complex are up modulated following both wavebands. The 500–550 nm region on the left is primarily responsible for the up-regulation of cell cycle progression and DNA repair and the 550–600 nm region is primarily responsible for the immune and inflammatory response. Both are regulated and controlled by the *IL1B* regulator which is the top modulated regulator in the liver FL response.

In zebrafish liver, FL exposure resulted in modulation of five pathways, with significant z-scores but relatively low gene coverage likely due to the lower numbers of liver DEGs. Characterizing the FL induced immune and inflammatory response, the APR pathway along with the Complement signaling, IL-1 mediated inhibition of RXR and the ILK and calcium signaling pathways were all up-modulated. Zebrafish liver modulated the APR pathway (2.83), ILK signaling (2.24), IL-1 mediated inhibition of RXR (2.01), and complement signaling (2.33) but in response to discrete waveband exposures (Fig. 3). In zebrafish liver, the primary stress responses signaled after FL exposure (i.e., APR, ILK and RXR signaling) were observed also modulated following exposure to the 500–550 nm waveband. The immune and inflammatory response increased in gene number and statistical significance following exposure to 550–600 nm light, as was the previously characterized complement signaling pathway response in FL. In addition, the upstream regulators could be segregated into these two 50 nm wavebands, with *IL1B* and *TNF* induction shown following both 500–550 and 550–600 nm, *IL8r* induction following 500–550 nm and *IFNG* induction following 550–600 nm light exposure (Fig. 6, Table S4). The second tier induced regulators include *IL6, Pkc, RAS, NFkb, PI3K* complex, and several others, that can be divided into these two discrete waveband exposures. In total, the waveband response in liver shows (a) an induced cell cycle proliferation and stress response following 500–550 nm, and (b) an induced inflammatory and immune response following 550–600 nm. These two wavebands are the most influential in the FL liver response as specifically traced through the waveband exposures.

### Waveband response summary

In total, zebrafish exposed to FL experience a highly conserved immune and inflammatory response in skin, brain and liver, as previously reported. This response can be traced to specific 50 nm wavelength regions in each organ. In skin, the immune and inflammatory response is induced following 400–500 nm, while in brain this response is induced upon exposure to 500–550 nm. Liver exhibits further red shifting of the inflammation/immune response to the 550–600 nm waveband. Each of these responses can be traced through the induction of specific pathways and genetic regulators to these discrete wavebands.

## Discussion

In skin, FL exposure served to modulate significantly more DEGs (3 fold more) than any single waveband region (Table 1). Fish skin is a complex organ consisting of multiple cell types (globule cells, immune cells, epithelial cells, etc.) including many different pigment cell types, such as micro and macro malanocytes, xanthophores and iridophors, to name a few^8,9^. These cell types all create the multitude of unique colors and pigment patters that distinguish the various fish species, including zebrafish. FL is a complex light source with varying wavelength amplitude peaks and valleys throughout the emission spectrum (Fig. 1b). While the overall genetic signature of FL exposure can be easily deduced^2^ the unique wavelengths within the complex emission spectrum that may be responsible for this signature are more difficult to dissect. Each wavelength, and varying wavelength combinations, interact with multiple cell types and receptor molecules to produce a genetic and biological response. Therefore, while a single wavelength might produce one genetic signature, the combination of that wavelength with another might mute, amplify or altogether change the genetic response^3^.

In liver and brain however the FL response was muted when compared to the waveband exposures (Table 1). Again, while this could be explained by antagonism (i.e., compensatory cancelation) between wavelengths in the broader spectrum FL light as previously observed in *Xiphophorus maculatus*^3^, it could also be that the effective dose of each wavelength in the FL spectrum is attenuated by penetration through the body. Even though each fish was given the same 35 kJ/m^2^ of FL or each respective waveband.

Following FL exposure, the primary response in skin, brain and liver of three commonly used biomedical models, zebrafish, medaka and hairless mouse, was an up modulation of *IL1B* regulation of APR pathway ultimately leading to increased immune and inflammatory responses^2^. To try and determine the wavelengths within the FL spectrum responsible for this effect, zebrafish were exposed to discrete 50 nm wavebands of light from 300–600 nm. Each waveband region was genetically characterized using IPA to determine what regulators, pathways and cellular functions were modulated. Following this analysis, zebrafish skin showed modulation of the APR pathway after 400–450 and 450–500 nm waveband exposures. In addition, other supporting pathways including GP6 signaling, Prothrombin activation, production of Nitric Oxide and ROS in macrophages and suppressed LXR/RXR activation were also modulated in these two wavebands as well as in FL exposed skin samples. These two waveband regions are firmly in the violet- blue region which is known to activate many biological molecules including carotenoids, FAD, cryptochromes and blue light photoreceptors^10–13^. The major FL response of up-regulated immune and inflammation appears due to a very small fraction of emission FL spectrum, although this region does include three intense mercury peaks at 404, 435 and 544 nm^14^; (Fig. 1b), It remains to be tested if these peaks have anything to do with the observed genetic modulation, however, the fact that APR up-modulation is also observed in the 450–500 nm waveband, well away from these mercury peaks, would seem to discount this possibility.

The key pathway modulated in all three zebrafish organs following FL exposure was the APR signaling pathway. This important pathway is known to be regulated by *IL1B* and *TNF* and stimulates cellular growth, stress, immune and inflammatory responses. Interestingly, this pathway was stimulated by wavebands from 400–600 nm in an organ and function specific manner. In skin, the APR was up-modulated in response between 400–500 nm. The 450–500 nm waveband also exhibits up-regulation of the osteoarthritis pathway, which is also observed following FL exposure, that indicates an increase in inflammation. Narrowing down the specific waveband that initiates the APR is likely more complicated in skin compared to other organs due to the cellular complexity of this organ. Zebrafish skin is comprised of three pigment cell types, melanophores, xanthrophores and iridophores and each fish has a different ratio of these three distinct populations^9,15,16^. Light is also absorbed and scattered differently in each cell type making expression of key light responsive elements variable and difficult to pinpoint. Melanophores for example are known to absorb all wavelengths of light giving them their black appearance; they are also known to stimulate and release proinflammatory cytokines, chemokines and growth factors such as *IL1*, *IL6*, *TNF*, *TGFb*, etc.^17^ that would be expected to activate the APR response. Melanin absorption is much higher at lower wavelengths, where the absorption at 400 nm is roughly twice that at 500 nm, and about one fourth that of 600 nm^18^. This is consistent with the observed induction of APR in the skin. Xanthrophores and iridiphores likewise have unique absorption and scattering wavelengths. Depending on the overall contribution of each cell type to the collected skin sample the amount of absorption and consequently the resulting signaling cascades activated by each waveband of light may be altered. Future single cell transcriptomic experiments performed after waveband exposures may resolve this question. However, this will require significant technological improvement as this technique has yet to be successfully applied to adult fish skin.

In brain, two regions, (i.e., 450–500 and 500–550 nm), invoke the FL induced immune and inflammatory responses, while the 300–350 nm waveband, containing UVB and UVA wavelengths, suppresses this response. The major FL response is induced by 500–550 nm waveband exposure in the brain. This waveband region is in the largest FL emission peak and is 50–100 nm red shifted from the observed skin specific waveband response, indicating brain may be directly absorbing light as these longer wavelengths may be expected to penetrate deeper into the body. While it is largely excepted that chromotaphores on the surface of skin absorb the light signal and pass the information to internal organs through signal transduction^19,20^ it has also been discovered that cultured zebrafish organs can be light entrained outside of the animal giving rise to the hypothesis that direct light reception by internal organs may be possible^21^. While both possibilities exist, it is interesting that in the brain a red shift occurs that coincides with stimulating transcription of *IL1B* and *TNF*, perhaps supporting direct light absorption by this organ. This also may suggest why longer wavelengths appear to be dominant to shorter ones in the ability to incite a genetic response. For example, we now know that in brain, 300–350 nm suppresses *IL1B* and the stress response, while 500–550 nm exposure stimulates the same gene sets; however, following FL exposure which contains all of these wavelength regions, the *IL1B* regulator is stimulated and the cellular stress response activated. Therefore, as observed in the *X. maculatus* skin, it seems that the longer wavelengths in the 500–550 nm stimulatory effect dominates the 300–350 nm suppression within the same pathways as previously observed for two wavebands exposed in succession (i.e., *X. maculatus* skin^3^).

Liver, like brain, shows a primary response and stimulation of *IL1B* by 500–550 and 550–600 nm. These further red shifted wavebands, compared to skin, support the penetration of longer wavelengths into the liver to elicit the immune and inflammation responses. The response in liver is split between the wavebands with 500–550 nm initiating the *IL1B* and *TNF* regulator, but more prominently used to stimulate cascades for cell growth and development such as *IL8r* and *PI3K complex* through ILK and Calcium signaling. However, following 550–600 nm exposure the *IL1B* and *TNF* regulators promote cellular stress regulators such as *IFNG* and *JAK/STAT* that can initiate a cellular stress response by up-regulation of the immune and inflammatory response through pathways such as complement and APR signaling. While *IL1B* and *TNF* show modulation by two different 50 nm waveband regions in liver, the specificity to selectively trigger one response (cell growth) versus another (cell stress) is surprising, and suggests these upstream regulators are not only light responsive but triggered by specific light wavelengths. Similar results have been previously demonstrated in *X. maculatus* skin whereby FL and a select 10 nm waveband (i.e, 520–530 nm) suppressed the *EGFR* pathway, while exposure to the 450–500 nm waveband selectively up-modulated this same pathway. Not only were the upstream regulators shown to be selectively and precisely controlled by specific wavebands of light, but this was the first indication that some wavelengths are dominant to others in activating or suppressing specific genetic pathways^3^. Likewise, in the brain dataset we see specific suppression following the lower waveband exposures with activation of the same pathways following the exposure to higher wavebands. In addition, the liver dataset shows *IL1B* modulated cellular growth is dominant to the stress response following 500–550 nm exposure; however, this same up-stream regulator selectively turns on the cellular stress response following exposure to 550–600 nm light. Collectively, these results indicate the overall genetic effects of FL exposure can be specifically assigned to relatively small visible wavelength regions and are different for each organ. Further, these results suggest specific waveband exposures of the intact animal may be employed to selectively alter the genetic state of specific organs in a pre-determined fashion, supporting similar conclusions in studies performed with other fish models^3^.

## Conclusions

The induced cellular immune and inflammatory responses observed following FL exposure of three vertebrate biomedical models; zebrafish, medaka and mouse, can be assigned to small wavelength regions in zebrafish skin, brain and liver.

The results indicate the direct light receiving organ, skin, induces a cellular immune and inflammatory response starting at 400 nm and peaking after 450–500 nm light, while the brain shows similar responses 50 nm red shifted (500–550 nm) and the liver 100 nm red shifted (i.e., 550–600 nm).

This highly conserved response observed in three organs indicates that while there is likely signal transduction from the eyes and skin, there is likely also direct reception within internal fish organs that serve to induce the immune and inflammatory responses observed following FL exposure.

Overall, these data support previous findings^3^ that specific waveband exposures of the intact animal may be employed to selectively alter the genetic state of select pathways within specific organs in a pre-determined fashion. However, the effects of waveband exposure must be empirically determined for each organ.

## Methods

### Fish utilized and FL exposure

Mature adult male *Danio rerio* (TU, zebrafish) were supplied by the Zebrafish International Resource Center in Eugene, OR. These zebrafish were acclimated to the same environment within the *Xiphophorus* Genetic Stock Center for 4 weeks prior to use in the described experiments. FL exposure (35 kJ/m^2^) was performed as previously detailed. All protocols were carried out as previously described^1,2^. Prior to light exposure, fish were placed individually into 100 mL of filtered home aquaria water and kept in the dark for 14 hrs. FL exposure occurred in UV-transparent cuvettes (9 cm × 7.5 cm × 1.5 cm) in 90 mL of water. The exposure cuvettes were suspended 10 cm between two banks of two (total of 4 lights) unfiltered 4,100 K fluorescent lights (Philips F 20T12/CW 20 watts, Alto) mounted horizontally on each side of a wooden box exposure chamber^1,2^. After FL exposure, all fish were returned to the dark in 100 mL filtered aquaria water for 6 hrs and then euthanized and dissected for RNA isolation. In addition, 3 fish were placed into the cuvettes following the procedure outlined above and placed into the exposure chamber, but with the lights turned off (sham treated fish). All other protocols described were also followed for the sham treated samples.

### Specific waveband exposure

For discrete waveband exposures of zebrafish to the six 50 nm wavebands between 300–600 nm (e.g., 300–350, 350–400, etc. to 600 nm), we utilized a TLS-300X Series Tunable Light Source (Newport Corporation, Irvine, CA, USA) containing an Ushio 300 W Xenon Short Arc Lamp Model 6258. Exposures were as detailed previously^3,4^ and each exposure received a total dose of 35 kJ/m^2^. Briefly, light emitted from the source was passed through a Cornerstone 130 Monochromator (Newport Corporation, Irvine, CA, USA) to define specific wavelengths. The bulb was burned in 15 min prior to all exposure treatments. The specific wavelengths were divided by 2 fiber optic light cables, allowing the fish to be exposed on both sides simultaneously to the defined wavelengths of light. Spectral distributions were made to determine the power output of each light source at specific wavelengths using a Newport 1918-R power meter (Newport Corporation, Irvine, CA, USA). The spectral distribution of the xenon light source was measured at full spectrum (0 nm) using an Ocean Optics STS 350–800 nm Microspectrometer (Ocean Optics Inc., Dundedin, FL, USA) and OceanView software v1.5 (http://oceanoptics.com/product/oceanview/). The microspectrometer was calibrated to a known standard using Ocean Optics Halogen Calibrated Light Source HL-3P-CAL (Ocean Optics Inc., Dundedin, FL, USA). To cover each wavelength in each 50 nm region, the monochromator was set to scan and repeat (i.e. loop) using Asoftech Automation (http://www.asoftech.com/) through the wavelengths of each region (1 nm/sec for 50 sec) for the duration of the light exposure (Table 3).

**Table 3:** The exposure time was adjusted accordingly to ensure each fish received 35 kJ/m^2^ of light.

Waveband Increment (nm)	Time (min)
300–350	42
350–400	35
400–450	30
450–500	31
500–550	37
550–600	47

Each waveband region was calibrated and the time was adjusted to ensure the same dose was given. The adjusted times are presented in minutes.

Each fish was then placed in a 4 cm long × 1 cm wide × 4.5 cm high quartz cuvette filled with 14 mL of filtered aquaria water. The cuvette was then centered between the 2 fiber optic light cables and covered by a box to eliminate ambient light. After exposure, the fish was removed from the cuvette, rinsed with filtered aquaria water, placed back into a 125 mL flask filled with 100 mL of filtered aquaria water, and placed in the dark for 6 hrs to allow for gene expression prior to sacrifice and organ dissection. Sham treated fish went through the same protocol as described above with the light source turned off ^3,4^.

### RNA isolation and sequencing

All organs were dissected into 300 μL RNA*later* (Life Technologies, Grand Island, NY, USA) and stored in the −80 °C freezer except for the skin samples which were immediately placed in 300 μL TRI Reagent (Sigma Inc., St Louis, MO, USA) and flash frozen in an ethanol dry ice bath. Total RNA was isolated from skin, brain and liver of zebrafish using a TRI Reagent (Sigma Inc., St Louis, MO, USA) chloroform extraction followed by the Qiagen RNeasy (Qiagen, Valencia, CA, USA) isolation protocol. Skin was homogenized in 600 μL TRI Reagent using a handheld tissue disruptor followed by addition of 120 µL of chloroform. Samples were vigorously shaken and then phases partitioned by centrifugation (12,000 × *g* for 15 min at 4 °C). After extraction, the RNA was precipitated with 500 μL 70% EtOH and further purified using a Qiagen RNeasy mini RNA kit following the manufacturer’s protocol. Residual DNA was eliminated with an on-column DNase treatment at 25 °C for 15 min. RNA quality was assessed with an Agilent 2100 bioanalyzer (Agilent Technologies, Santa Clara, CA), and quantified with a Qubit 2.0 fluorometer (Life Technologies, Grand Island, NY, USA). All samples sent for sequencing had RIN scores ≥ 8.0^1,2^.

### Differentially expressed gene (DEG) analysis

RNA sequencing was performed on libraries constructed using the Illumina TrueSeq library preparation system that employs polyA selection. RNA libraries were sequenced as 100 bp paired-end fragments using an Illumina Hi-Seq 2000 system (Illumina, Inc., San Diego, CA, USA). From 39–108 million raw reads were generated for the RNA samples post-filtration. All raw reads were subsequently truncated to remove library adaptor sequences using a custom Perl script and short reads were filtered based on quality scores using a custom filtration algorithm that removed low-scoring sections of each read and preserved the longest remaining fragment^22^. Filtered reads were mapped using Tophat2^23^ to the *Danio rerio* (zv9) genome. The percentage of reads mapped were assessed by Tophat2, and sequencing depth assessed by SAMtools depth, respectively (Table 4)^23,24^. Gene expression was assessed by featureCounts using genome annotation from Ensembl database v79^25^, and differentially modulated genes were determined using the R-Bioconductor (www.bioconductor.org) package edgeR^26^ with a |log_2_(fold change)| ≥ 1.0 (FDR < 0.05), (Table 1).

**Table 4:** Read depth and RNA-Seq statistics for FL exposed, sham and specific waveband treated zebrafish samples.

Species	Organ	Filtered Reads (×10^6^)	Read Length (×10^9^)	Reads Mapped (×10^6^)	Reads Mapped (%)	Coverage (x)
Zebrafish FL	Skin	67.0	7.3	54.6	81.5	120.9
		71.5	7.8	58.1	81.2	129.4
	Brain	51.0	6.0	40.3	79.0	99.6
		51.4	6.0	40.5	78.7	99.5
	Liver	57.7	6.9	48.7	84.4	113.5
		47.8	5.6	40.6	85.0	93.1
Zebrafish FL Sham	Skin	78.7	8.6	64.7	82.2	142.7
		75.0	8.2	61.4	81.8	135.7
	Brain	54.9	6.4	43.3	78.7	106.0
		55.1	6.4	43.5	78.9	106.5
	Liver	47.2	5.6	39.8	84.4	91.9
		48.9	5.8	41.5	84.9	95.3
Zebrafish 300–350	Skin	74.2	11.1	60.1	80.9	125.8
		89.3	13.4	72.5	81.3	151.9
	Brain	85.5	12.8	70.8	82.8	148.3
		84.4	12.7	69.0	81.7	144.5
	Liver	96.1	14.4	79.5	82.7	166.4
		112.8	16.9	96.0	85.1	200.9
Zebrafish 350–400	Skin	96.6	14.5	80.4	83.3	168.4
		94.5	14.2	77.6	82.1	162.5
	Brain	86.5	13.0	71.9	83.1	150.6
		98.9	14.8	82.0	82.9	171.6
	Liver	85.5	12.8	71.4	83.5	149.5
		90.5	13.6	76.7	84.8	160.7
Zebrafish 400–450	Skin	92.2	13.8	75.8	82.3	158.8
		81.7	12.3	66.4	81.2	139.0
	Brain	100.6	15.1	83.1	82.6	174.0
		107.7	16.2	89.7	83.3	187.9
	Liver	127.6	19.1	108.8	85.3	227.9
		97.3	14.6	81.8	84.1	171.3
Zebrafish 450–500	Skin	94.4	14.2	76.3	80.8	159.7
		80.1	12.0	64.9	81.0	135.9
	Brain	96.6	14.5	80.4	83.3	168.4
		88.3	13.3	73.6	83.3	154.0
	Liver	94.8	14.2	79.7	84.1	167.0
		90.7	13.6	74.9	82.6	156.9
Zebrafish 500–550	Skin	87.0	13.0	72.1	82.9	151.0
		104.3	15.6	84.0	80.5	175.8
	Brain	85.3	12.8	70.9	83.1	148.4
		87.9	13.2	72.3	82.2	151.3
	Liver	93.3	14.0	78.5	84.1	164.3
		108.0	16.2	80.3	74.4	168.2
Zebrafish 550–600	Skin	80.3	12.0	65.4	81.4	136.9
		85.9	12.9	70.1	81.6	146.7
	Brain	99	14.8	81.8	82.7	171.4
		94.8	14.2	78.3	82.6	163.9
	Liver	86.1	12.9	70.2	81.5	146.9
		110.2	16.5	94.5	85.7	197.8
Zebrafish Waveband Sham	Skin	84.9	12.7	69.1	81.4	144.8
		97.2	14.6	79.0	81.2	165.4
	Brain	97.4	14.6	80.8	83.0	169.3
		98.6	14.8	81.7	82.8	171.1
	Liver	106.6	16.0	89.9	84.4	188.3
		93.1	14.0	75.4	81.0	157.9

Genes identified as being differentially modulated in response to FL or due to 50 nm waveband exposure, were further analyzed for organ specificity using InteractiVenn^27^ and for functional specificity with Ingenuity Pathway Analysis (IPA, Qiagen, Redwood City, CA). IPA-based gene expression analysis yielded gene clusters, genetic pathways, functional classes, and potential up-stream regulators to aid in mechanistic interpretation. As previously described^1,2^, the term “pathways” is short for canonical pathways assigned by IPA based on the light exposure input DEG data. In IPA, known pathways are drawn as pictures with input DEGs overlaid onto them that are identified by symbols and colors indicating known functions and direction of modulation. A z-score algorithm is used to determine if a pathway is up or down regulated based on the genes that fall into that particular pathway and the direction of modulation. IPA assignment of DEGs into “functions” or “functional classes” relates the input DEGs to known disease states and biological functions as published in the scientific literature. Functional classes are visualizations of the biological trends in the light effected DEG dataset and may be used to predict the effect of gene expression changes of the entire dataset on biological processes and known cellular functions. Functional assignment uses an algorithm to assess the dataset as a whole and predict what is collectively occurring on a larger down-stream scale.

### Validation of RNA-Seq gene expression results

NanoString (NanoString Technologies, Inc., Seattle, WA), with a custom panel for zebrafish (Table S1) was used as an independent technology to confirm the DEGs identified using RNA-Seq. Aliquots of the RNA (500 ng) used for RNA-Seq were also used for the NanoString nCounter assay. Hybridization protocols were strictly followed according to manufacturer’s instructions^28^. Samples were hybridized overnight at 65 °C with custom probes and transferred to the NanoString Prep Station. The NanoString cartridge containing the hybridized samples was immediately evaluated with the NanoString nCounter based on unique color-coded signals. Probe counts were quantified through direct counting with the nCounter Digital Analyzer. Data analysis was performed by lane normalization using a set of standard NanoString probes followed by sample normalization using a set of 10 housekeeping genes. Fold changes were calculated on normalized counts and plotted using Microsoft Excel^1,2^.

All RNA-Seq short read sequence data utilized to prepare the differential expression analyses presented herein are deposited on the *Xiphophorus* Genetic Stock Center website (https://www.xiphophorus.txstate.edu) and will be made available upon reasonable request to the corresponding author. The final differential expression gene lists are published with the supplementary data files associated with this manuscript.

### Ethics declaration

All animal studies were approved by the Texas State University Institutional Animal Care and Use Review Board (IACUC protocol #20173294956). All fish used in this study were from aquaria housed stock and were kept and sampled in accordance with the applicable national and international legislation regulations governing animal experimentation.

Only fish, *Danio rerio* (TU, zebrafish) that were supplied by the Zebrafish International Resource Center in Eugene, OR were utilized in these studies. These animals were maintained in the *Xiphophorus* Genetic Stock Center (http://www.xiphophorus.txstate.edu/), a scientific animal resource facility supported by the National Institutes of Health. The fish utilized were raised from laboratory stocks and maintained in accordance with the applicable OLAW guidelines governing animal experimentation in the USA.

## Supplementary information

Supplementary Figure.

Supplementary Table 1.

Supplementary Table 2.

Supplementary Table 3.

Supplementary Table 4.

Supplementary Legends

## Data Availability

The datasets generated during and analyzed during the current study are available from the corresponding author on reasonable request.
