# Supplementary material for: Deconvoluting Wavelengths Leading to Fluorescent Light Induced Inflammation and Cellular Stress in Zebrafish (Danio rerio)
Source: Sci Rep. 2020 Feb 24;10:3321. doi: 10.1038/s41598-020-59502-5 (PMC7039929; doi:10.1038/s41598-020-59502-5)
Supplement: Supplementary file 6 — Supplementary Legends [file 41598_2020_59502_MOESM6_ESM.pdf]

Supplemental Figure Legends:

Figure S1: FL modulated pathways in zebrafish skin, brain and liver consistent with an up-regulation of the immune and inflammatory response. 50 nm regions were surveyed from 300-600 nm to determine which specific wavelengths were responsible for this response. Red indicates specific canonical pathways as determined by IPA that had a z-score of > 2 and green represent pathways with a z-score < 2. The numbers inside of each box represent the unique number of genes represented by each pathway. Both 400-450 and 450-500 nm reflected the FL response in skin; 500-550 nm in brain and 550-600 nm in liver. The primary waveband mimicking the FL response is highlighted in blue for each organ.

Supplemental Table Legends:

Table S1: A custom NanoString panel was used for all RNA-Seq verification. Panel selected genes and housekeepers are listed along with oligo sequences.

Table S2: IPA predicted upstream regulators in response to FL and each waveband exposure in zebrafish skin.

Table S3: IPA predicted upstream regulators in response to FL and each waveband exposure in zebrafish brain.

Table S4: IPA predicted upstream regulators in response to FL and each waveband exposure in zebrafish liver.
